# Attitudes and avatars instrument: development and initial testing

**DOI:** 10.1186/s12889-015-2019-4

**Published:** 2015-07-14

**Authors:** Annmarie A. Lyles, Susan K. Riesch, Roger L. Brown

**Affiliations:** College of Nursing & Health Innovation, Arizona State University, Phoenix, Arizona USA; School of Nursing, University of Wisconsin-Madison, Madison, Wisconsin USA

**Keywords:** Adolescent boys, Weight, Instrument development, Perceptions, Avatars

## Abstract

**Background:**

The purpose of the study was to develop and test the initial psychometric properties of the ATTitudes and Avatars INstrument (ATTAIN). The integrated behavior model guided instrument development to measure the young adolescent boys’ attitudes, intentions and actions to change their bodies.

**Methods:**

An adolescent health expert panel and young adolescent boys were recruited to test for content validity. Fifty-nine boys 11 to 14 years of age were recruited at a middle school in the USA during physical education class to conduct a pilot study to test for internal consistency and test-retest reliability.

**Results:**

The ATTAIN was found to have high content validity, slightly below recommended levels for internal consistency, and varied test-retest reliability.

**Conclusion:**

The long-term goal of the development and testing of the ATTAIN is to make it available to researchers and professionals to screen and focus on adolescents’ perceptions of their bodies and using those perceptions to attain and maintain healthy bodies. The results of this study suggest preliminarily a theoretically derived instrument with appropriate content for young adolescent boys and variable reliability. The attitudes, intentions, and actions survey items and avatars as measured by the ATTAIN, were meaningful to the boys. The ATTAIN has potential to be used as a screening instrument for young adolescents boys and understanding their attitudes toward their bodies; however, it will require continued development and testing to establish construct and discriminant validity.

## Attitudes and avatars instrument: development and initial testing

Body mass index (BMI) is limited to body weight and as such is an incomplete measure for appropriate referral and follow up for adolescent weight issues. A theoretically derived, reliable, and valid instrument that captures adolescents’ perceptions of their weight, bodies, and body parts is needed to augment weight screening. The ATTitudes and Avatars INstrument (ATTAIN) was designed to measure young adolescent boys’ attitudes toward their bodies within the context of the integrated behavior model [1] and to document perception of their current and preferred bodies through the creation of computerized selves (avatars). The purpose of this paper is to describe the development and initial testing of the ATTAIN.

## Background

Over the past 30 years, childhood obesity has tripled, with more than one-third of children and adolescents in 2008 being overweight or obese [2, 3]. The prevalence of above-normal body weight in adolescents is a vexing and complex public health problem in the United States and internationally. Ogden and colleagues [3] found from 2009–2010 the prevalence of obesity higher among adolescents than preschool-aged children (18.4 % ages 12–19, 18 % ages 6–11, 12.1 % ages 2–5) and higher among boys than among girls (18.6 % of boys, 15 % of girls).

Overweight and obese adolescents can face emotional and physical challenges that can affect their learning. In cross-sectional studies, overweight and obese adolescents had lower cognitive functioning than healthy weight adolescents [4, 5]. In addition to obese and overweight adolescents not learning to their full potential there is a likelihood of them becoming obese adults [6, 7] putting them at risk for adult health problems such as heart disease, type 2 diabetes, stroke, cancer, and osteoarthritis [8]. More research is needed to identify the behavioral, biological, and environmental factors sustaining this problem. One approach to confront childhood overweight and obesity is to examine how perceptions of weight are related to one’s intention and action to change or maintain weight and make the assessment of his/her perception part of routine screening and referral procedures [9].

BMI is a common, inexpensive measure used to screen adolescents who are overweight and obese and can be plotted on the Centers for Disease Control (CDC) BMI-for-age growth charts [10] to obtain a percentile ranking. The relationship between BMI and body fat, cardiovascular risk factors and long-term health outcomes has considerable individual variability, making BMI an imperfect proxy for obesity [11].

A more thorough screening for weight problems, referral, and follow up should include assessing youth’s perceptions of their bodies, body parts, and weight. Interventions that focus on preventing obesity may have limited success due to misperception of overweight [12]. One of the chief barriers to effective treatment of weight issues is lack of perception that weight is a problem [13]. We need to understand youth’s perception of their bodies so to screen and refer them appropriately [9]. Perception influences how intensely an individual engages in behavior change, [14] and misperception awareness should be incorporated as a component of obesity prevention. Youth are more likely to adopt healthy lifestyle behaviors if they recognize themselves as overweight [12].

According to Cohane and Pope, [15] most investigators use questionnaires and figure drawings to examine body image and attitudes in children. Using only one or two questions to assess weight perceptions such as “How do you describe your weight?” or “Do you think you are thin, about right, fat, too fat?” [16] may not provide consistency in response and lead to perceptions being inaccurate, invalid, and unreliable. Figure drawings typically include sets of seven to nine male and/or female figure drawings, ranging from extremely thin to extremely fat, which have been adapted from those created by Stunkard, Sorenson, and Schlusinger [17]. Al-Sendi and colleagues [16] suggested nine figure silhouettes as too limited in choices for an adolescent to reflect what the body looks like (current and preferred). Further, the silhouettes increase in size by the whole body not by individual body parts. Adolescents may perceive they have a bigger abdomen but smaller legs and arms or bigger legs but a smaller abdomen; the silhouettes do not accurately reflect their perceptions of their current and ideal bodies. Thus, an instrument is needed that allows the adolescents to manipulate different body parts to reveal accurate perceptions. Developing avatars to reflect these perceptions that is guided by theory is the intent of the ATTAIN.

### Integrated behavior model

The integrated behavior model (IBM) [1] is built upon constructs from the theory of reasoned action and theory of planned behavior and proposes that an important determinant of behavior is intention to perform the behavior. A person is not likely to carry out (action) a recommended behavior without motivation (intention). The behavioral intention is determined by three constructs: attitude, perceived norm, and personal agency. Findings of an umbrella review suggest that intention and sedentary behavior have the strongest evidence base as determinants of healthy and unhealthy behavior among youth [18]. This paper will focus on the construct of attitude.

Attitude is defined by Montano and Kasprzyk [1] as a person’s overall favorableness or unfavorableness toward performing the behavior and includes boys’ feelings and beliefs expressed as their satisfaction or dissatisfaction with weight. Boys generally display less overall concern than girls do; however, boys of all ages report dissatisfaction with their bodies [15] Overweight girls have been found to be more dissatisfied than overweight boys [19]. Body satisfaction in adolescent boys may be associated with gaining muscle, whereas dissatisfaction may be associated with gaining fat. Adolescents of the same height and age can have the same BMI percentile as a result of an increase of body weight from gaining either muscle or fat [20]. Instead of being a motivator for engaging in healthy weight management, however, lower body satisfaction is associated with the use of unhealthy behaviors that may lead to weight gain and poorer overall health [21–23]. Beliefs can include how accurate adolescents are when classifying their weight category. If adolescents’ beliefs about their weight are not accurate, health professionals’ discussions about their weight may not be consistent with the adolescents’ perceptions.

Attitudes in part determine the intention that leads to action to carry out the behavior. Adolescents may have taken action to lose weight in the past and not obtained their goals. Their lack of intention to take an action due to their lack of success or frustration may have prevented the adolescent from changing behaviors [14]. Bittner and colleagues found the overweight sample, when compared with the obese sample, was less accurate with their perception of weight, less intent to lose weight, and less likely to take action to lose weight [14]. The overweight group may not be visibly overweight, or recognized as having a high BMI and thus missed the opportunity to intervene [14]. Their intentions to take action may be delayed or never generated due to inaccurate perceptions of their bodies. A potential benefit for future research is for adolescents to create current, preferred, and actual perceptions of their bodies that can be used to assess their attitudes (satisfaction with their body parts and accurate perceptions).

### Purpose and aims

The purpose of the study was to develop and test the initial psychometric properties of the ATTitudes and Avatars INstrument (ATTAIN). The ATTAIN includes survey items and avatar creation. Procedures for instrument development as outlined by DeVillis [24] were implemented. The study consisted of three steps: (1) develop the avatars; (2) test for content validity; and (3) test for internal consistency and test-retest reliability.

### Step 1. avatar development

The vision for the avatars was for boys to be able to manipulate different body parts (head, neck, upper arm, forearm, chest, waist, hips, thigh, and calf) of avatars onscreen to create current and preferred representations. The idea was that the boys could manipulate each avatar to make each body part bigger or smaller to reflect their perceptions of how their bodies currently look (Current Avatar) and how they prefer their bodies to look (Preferred Avatar).

**Fig. 1:**
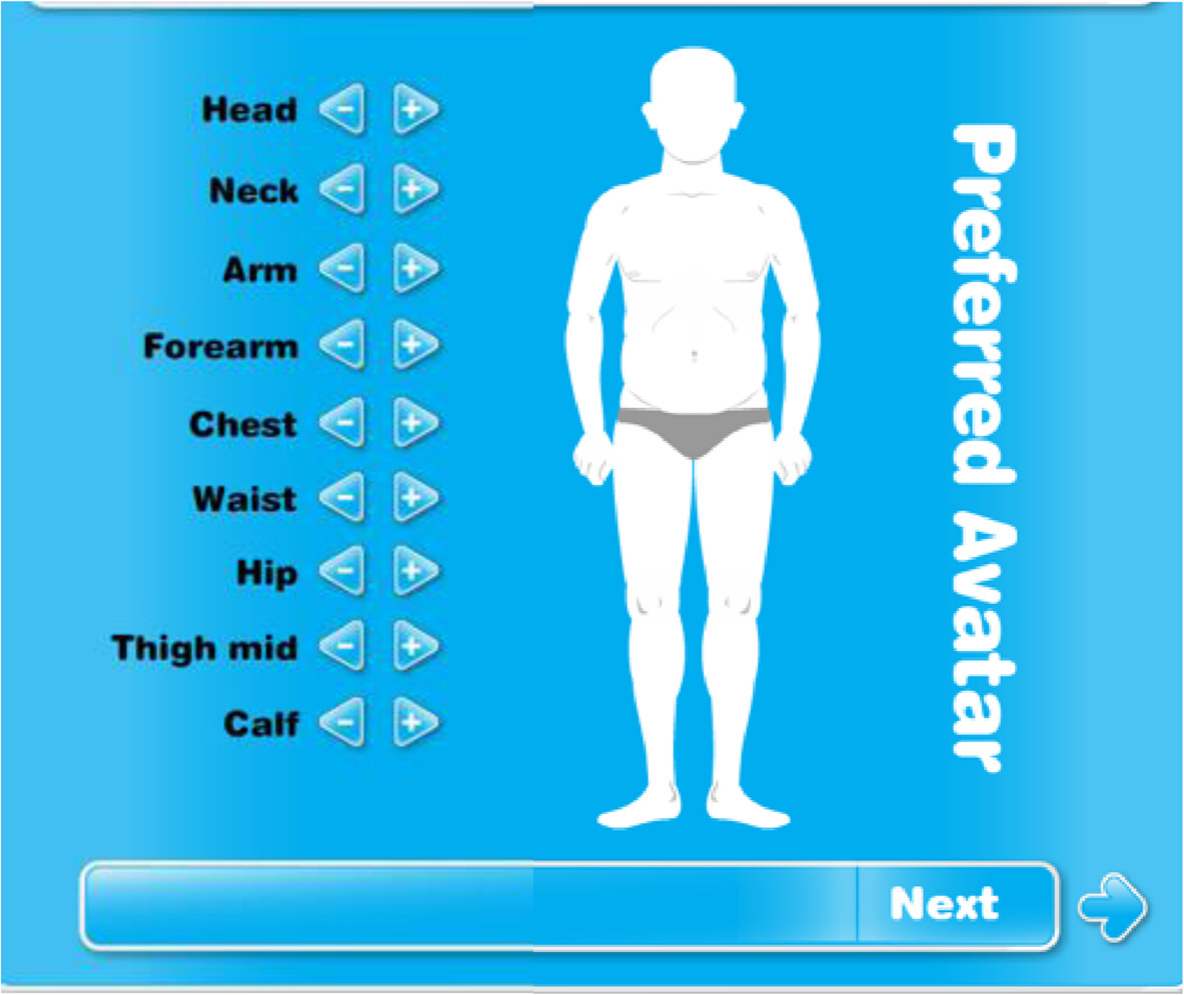
Avatar example

Height was the anchor used to generate a proportional avatar on the screen for the boy to manipulate. The 50th percentile measurement for each height for boys 11 to 14 years of age was the standard used for each initial avatar. The measurements for each body part were configured using anthropometric body measurements of 977 adolescent boys 11 to 14 years of age as reported by McDowell et al., [25] which included height, weight, head, mid upper arm, waist, thigh, and calf circumferences. The neck, chest, forearm, and hip measurements for the 50th percentile were estimated using boys’ sizing charts [26]. An example of an avatar is displayed in Fig. 1.

### Step 2. content validity

All study procedures were reviewed and approved by the University Health Sciences Institutional Review Board. Prior to the development of the survey items, the researcher conducted semi-structured interviews with 20 adolescent boys ages 11 to 14 to understand what boys thought about changing their weight; what they liked and disliked about their bodies; if they felt they were in charge of their weight; if they thought weight was important; if weight was something that was discussed among the boys and their parents, friends, or others in their lives; and what they wanted to do about their weight [27]. These thoughts were included in the development of the survey items.

## Methods

### Sample

Two samples assessed the content validity of the survey items: (a) a panel of six health professionals whose specialties were adolescent health and obesity and (b) six adolescent boys. The researcher knew the health professionals and boys who agreed to participate.

### Procedure

The adolescent health experts were provided with instructions on how to evaluate the ATTAIN. The instructions included a brief summary of the ATTAIN; definition of attitudes; and a definition of the content domain of perception. Attitude was defined as “overall favorableness or unfavorableness toward their body, body parts, and weight and included satisfaction or dissatisfaction with their bodies, whether it is important to change their bodies, and accuracy or inaccuracy of their perceptions”. Perception was defined as “a way of regarding, understanding, or interpreting something, in this case, one’s self including body, body parts, and weight”. Their instructions included a 4-point individual item content validity index (I-CVI) [28, 29]. On this index, the experts indicated the degree to which the items were representative of the attitude construct and clear by indicating: *strongly disagree*, *disagree*, *agree*, and *strongly agree*. The I-CVI is the proportion of those experts who rated representativeness of *agree or strongly agree* for each attitude item. With every item, the expert had the option of providing feedback on how to make the item clearer and to provide global feedback about comprehensiveness and instruction clarity.

Boys 11 to 14 years of age and varying weights were asked in individual interviews to review each ATTAIN item and report whether the items were understandable, important to them, and appropriate. The boys were asked to read the survey items as though boys their age and between 11 and 14 years of age were taking the survey, but also to write down what did not make sense, suggestions on how to make the items clearer, and if items needed to be added or deleted. Lastly the boys were asked to provide feedback about user friendliness.

### Data analysis

A content validity of individual item (I-CVI) as well as the whole scale (S-CVI) for the health experts was calculated. It was decided *a priori* that those items without the recommended content validity index of 0.78 be eliminated or revised [28, 29]. The individual comments from the adolescent boys were documented. The research team critically evaluated the feedback provided by the experts and adolescent boys to make changes to the instrument.

## Results

The ATTAIN demonstrated high content validity. The health professionals had an I-CVI of 0.83 on one attitude item and an I-CVI of 1.00 on the other 19 attitude items and avatars. For the intentions/actions there was an I-CVI of 1.00 on two items and an I-CVI of 0.83 on one item. With respect to clarity of items, there was an I-CVI of 0.66 on two items, an I-CVI of 0.83 on eight items, and an I-CVI of 1.00 on 13 items. For clarity of the avatar section there was an I-CVI of 1.00. All experts agreed (S-CVI = 1.00) the instrument was comprehensive and the instructions, rating scales, and formatting were clear. The attitude items that were not clear were revised according to the feedback given by the health experts. No additional items were added or deleted after the analysis.

The boys who were interviewed indicated that the ATTAIN was easy to use and expressed how much they enjoyed creating the avatars of themselves. All the boys reported it took 30 min or less to review all the items and create the avatars. The boys reported all the items were important and did not suggest any item be added or deleted.

### Step 3. reliability

#### Methods

All study procedures were reviewed and approved by the University Health Sciences Institutional Review Board.

##### Sample

The ATTAIN was tested with 59 boys 11 to 14 years of age. These boys were not sampled in any previous part of the instrument development. The inclusion criteria for the youth were (a) male gender; (b) between ages of 11 and 14; and (c) able to speak, read, and write English. Parents/guardians needed to be able to read and write English to provide consent. Subject exclusion criterion was cognitive disabilities that would limit understanding of the ATTAIN, as assessed by the physical education (PE) teachers.

##### Instrument

ATTAIN consisted of avatar creation and survey items. There were 20 survey items to measure the boys’ attitudes about their body, body parts, and weight. The items that reflected the boys’ attitudes incorporated what they thought of the size of the body parts, including their head, chest, stomach, arms, butt, and legs/thighs. Participants could choose a response of *too small, just right, too big,* and *I do not want to answer*. There were also items asking if they liked these body parts, if they thought it was important to change these body parts, and if they were a good weight for their height*.* Their response choices were *strongly disagree, disagree, agree, or strongly agree*. The last attitude item asked how they described their weight. They could respond with *underweight, about-right weight, little bit overweight, overweight, or very overweight.* Three items were included to measure intentions/actions about the ways the boys were trying to change their body compositions, what they were doing to change their bodies, and the reason for changing their bodies.

##### Recruitment

The recruitment plan consisted of meeting with the school personnel of a Midwestern rural middle school in the USA to discuss recruitment strategies. The study was introduced during the PE classes at the middle school. Study packets, consisting of a letter summarizing the study, the items included in the ATTAIN, parent consent, and boy assent, were distributed for the boy to take home and discuss with his parent(s). Consents and assents were returned to the PE teachers.

The boys, as part of a separate research grant acquired by staff at the middle school, had their height measured with a stadiometer and weight measured with a digital scale. The PE teachers measured the students with their PE dress of a t-shirt and shorts and provided the height and weight measurements to the investigator for those who assented to be in the study.

##### Data collection

On the day of data collection, all boys who assented were excused from PE class and chaperoned by the student research assistant to the school library where laptops were set up with the ATTAIN application. Each boy sat at a laptop with another laptop station separating each boy to maintain privacy during the completion of the ATTAIN.

The completion of the ATTAIN included the following: (a) a hyperlink to the instrument; (b) a login and password for each session; and (c) the actual completion of the survey, in which the boy entered his first name, avatar name, birthdate, height, ethnicity, race, and responses to the 23 survey items. Following the 23 items, each boy created a Current Avatar and a Preferred Avatar. The boys clicked on left and right arrows for each body part to make the avatars’ body parts smaller (left arrow) and bigger (right arrow). Each click on the arrow, whether bigger or smaller, represented one pixel or one centimeter of movement. The boy was able to make as many changes to the body parts as he wanted until the last screen where he logged out. Physical measurements of each boy were taken to compare with the data of each body part exported from the avatars. Measurements of the body parts included the head, neck, upper arm, forearm, chest, waist, hips, thigh, and calf. Though technically not an avatar, staying with the theme, the term Actual Avatar will be used to identify these body part measurements as a whole and individually and for making comparisons with the Current Avatar and Preferred Avatar.

For the Actual Avatar, each boy had his body parts measured twice using an anthropometric tape measure. Each research team member was trained using the measurement procedure developed based on recommendations by McDowell and colleagues [25] and the American College of Sports Medicine [30]. Each boy had one layer of clothing on (shorts and a shirt) and was measured in a separate room of the library with the door closed.

Following the measurements, the boy had the option of entering his name in a drawing for one of two iPod Touches. The boys who assented were reminded that they would complete the same procedure in 2 weeks without having their measurements taken.

##### Test-retest procedure

Those who assented completed the ATTAIN 2 weeks later to evaluate test-retest reliability [29]. On the day of data collection, the boys who assented were excused from PE class and chaperoned by the student research assistant to the school library where laptops were set up with the ATTAIN application. All steps identical to the first data collection episode were repeated, except no physical measurements were taken. Following the test-retest the boys could enter a drawing for an iPod Nano.

##### Data analysis

Statistical tests were completed using *Statistical Package for Social Science* (SPSS) software, version 21. Descriptive statistics with distribution frequency were used to describe the sample characteristics and survey items. Cronbach’s alpha was used to calculate internal consistency. Test-retest reliability for the individual items, overall scale, and avatars was quantified using intraclass correlation coefficient (ICC) [31]. The ICC (A,k) values were derived from a two-way random effects analysis of variance model [32]. Values for reliability greater than 0.70 would be considered acceptable [33].

### Results

Of the 335 packets distributed, 59 were returned with signed parental consents and participant assents, for a return rate of 17.6 %. All 59 boys completed the ATTAIN and had their body parts measured. For the test re-test two weeks later, 59 boys had signed parental consent and signed participant assent to complete the ATTAIN a second time. Of the 59 boys, 55 completed the ATTAIN for a second time; 4 boys were absent from class that day.

The boys’ demographics and height, weight, and BMI are displayed in Table 1. Almost half were age 13 (*M* = 12.6, *SD* = 0.83). Over three-quarters (78 %) self-reported their race as Caucasian/White, and nearly all (89.9 %) reported they were not of Hispanic ethnicity. In inches, the minimum measured height was 57 in. and maximum 72 in. (*M* = 63.4, *SD* = 3.94). The minimum measured weight in pounds was 69 lb and the maximum 291 lb (*M* = 122.7, *SD* = 36.65). Using the CDC BMI Percentile Calculator for Child and Teen, [10] the BMI percentile was calculated and a weight category assigned from the following weight categories: underweight (<5th percentile), healthy weight (5th percentile up to 85th percentile), overweight (85th percentile to less than 95th percentile), obese (95th percentile to less than 97th percentile), and high BMI (≥97th percentile) [3]. More than half of the boys were categorized as healthy weight (64.4 %), and there were no boys categorized as underweight.

**Table 1:** Demographics of sample as a result of pilot testing

Demographics						
Age in years	11 N (%)	12 N (%)	13 N (%)	14 N (%)	Mean	SD
	5 (8.5)	20 (33.9)	26 (44.1)	8 (13.6)	12.6	0.83
Race	White N (%)	Black N (%)	Asian N (%)	Native American N (%)	White & Native American N (%)	No answer N (%)
	46 (78)	1 (1.7)	3 (5.1)	0	2 (3.4)	7 (11.9)
Ethnicity	Hispanic N (%)	Non-Hispanic N (%)	No answer N (%)			
	6 (10.2)	53 (89.8)	0			
	Minimum	Maximum	Mean	SD		
Height	57 in.	72 in.	63.40	3.94		
	145 cm	183 cm	160.96	9.97		
Weight	69 lb	291 lb	122.69	36.65		
	31 kg	132 kg	55.65	16.62		
CDC BMI-for-age Percentile	6th	99th				
Weight Category	Underweight N (%)	Healthy N (%)	Overweight N (%)	Obese N (%)	High BMI N (%)	
	0	38 (64.4)	11 (18.6)	6 (10.2)	4 (6.8)	

#### Attitude

The frequencies of the attitude survey items are displayed in Table 2. The boys were asked their feelings and beliefs about their body parts. Most boys thought their body parts were *just right*. However, some boys thought their stomach was *too big*, arms *too small*, and thighs *too big*. In addition, the boys were asked if they liked their body parts. Most of the boys agreed they liked their body parts. Body parts they disagreed they liked were the stomach, chest, arms, and legs/thighs.

When asked if it was important to change their body parts, the same proportions of boys reported they disagreed as agreed it was important to change their chest and legs/thighs. However, a higher proportion of boys agreed it was important to change their stomach and arms. Most boys disagreed it was important to change their butt.

When asked how the boys perceived their weight, over half indicated they were *about right weight,* about three- quarters agreed they were a good weight for their height, and almost one-quarter indicated they were a *little bit overweight*. Even though no boys were classified as underweight, almost one-tenth described their weight as *underweight*. When comparing their weight description responses to their BMI-for-age weight category, more than half were accurate, over one-quarter underestimated, and almost one-fifth overestimated their description of their weight.

**Table 2:** Frequencies, means, and standard deviations survey items

Item				Response	Choices with	Coding	
	Mean	SD	Too small =1	Just right = 2	Too big = 3	No answer	
			N (%)	N (%)	N (%)	N (%)	
*My…*							
Head/neck is	2.02	0.13	0 (0.0)	58 (98.3)	1 (1.7)	0 (0.0)	
Chest is	1.97	0.52	9 (15.3)	4 (72.9)	7 (11.9)	0 (0.0)	
Stomach is	2.36	0.58	3 (5.1)	31 (52.5)	24 (40.7)	1 (1.7)	
Arms are	1.72	0.49	17 (28.8)	40 (67.8)	1 (1.7)	1 (1.7)	
Butt is	2	0.28	2 (3.4)	49 (83.1)	2 (3.4)	6 (10.2)	
Legs/thigh are	2.20	0.48	2 (3.4)	41 (69.5)	13 (22.0)	3 (5.1)	
			Strongly disagree = 1	Disagree = 2	Agree = 3	Strongly agree = 4	No answer
			N (%)	N (%)	N (%)	N (%)	N (%)
*I like my…*							
Head/neck	3.19	0.57	0 (0.0)	5 (8.5)	38 (64.4)	16 (27.1)	0 (0.0)
Chest	2.92	0.62	0 (0.0)	14 (23.7)	36 (61.0)	9 (15.3)	0 (0.0)
Stomach	2.69	0.73	3 (5.1)	18 (30.5)	31 (52.5)	6 (10.2)	1 (1.7)
Arms	3	0.73	1 (1.7)	12 (20.3)	31 (52.5)	14 (23.7)	1 (1.7)
Butt	3.07	0.50	0 (0.0)	5 (8.5)	42 (71.2)	9 (15.8)	3 (5.1)
Legs/thighs	3.09	0.71	0 (0.0)	12 (20.3)	29 (49.2)	17 (28.8)	1 (1.7)
*It is important for me to change my…*							
Head/ neck	1.93	0.53	10 (16.9)	42 (71.2)	6 (10.2)	0 (0.0)	1 (1.7)
Chest	2.53	0.77	4 (6.8)	26 (44.1)	23 (39.0)	6 (10.2)	0 (0.0)
Stomach	2.83	0.97	6 (10.2)	15 (25.4)	21 (35.6)	17 (28.8)	0 (0.0)
Arms	2.64	0.80	5 (8.5)	18 (30.5)	29 (49.2)	7 (11.9)	0 (0.0)
Butt	1.95	0.68	13 (22.0)	33 (55.9)	8 (13.6)	1 (1.7)	4 (6.8)
Legs/thighs	2.45	0.94	10 (16.9)	20 (33.9)	20 (33.9)	8 (13.6)	1 (1.7)
*I am a good weight for my height…*	2.90	0.71	1 (1.7)	15 (25.4)	32(54.2)	11 (18.6)	0 (0.00
*When you look at yourself, how do you describe your weight?*			Underweight = 1	About right weight = 2	Little bit overweight = 3	Overweight = 4	Very overweight = 5
	2.44	0.91	5 (8.5)	33 (55.9)	13 (22.0)	6 (10.2)	2 (3.4)

#### Intentions and actions

**Fig. 2:**
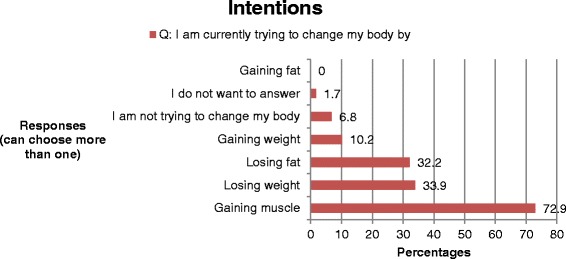
Adolescent boys’ response to their intentions to change their bodies

**Fig. 3:**
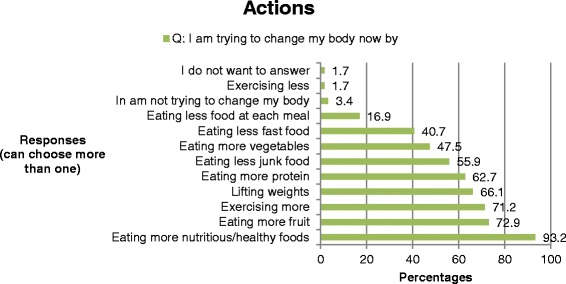
Adolescent boys’ responses to reasons to change their bodies

**Fig. 4:**
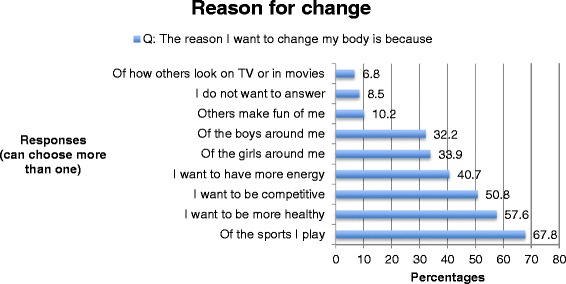
Adolescent boys’ responses to actions taken to change their bodies

The boys were asked about trying to change their bodies and had the option of choosing more than one response. Almost three-fourths of the boys were currently trying to change their body by *gaining muscle* (Fig. 2) by taking the action of *eating more nutritious/healthy foods* (Fig. 3). Over a half wanted to change their body because of *the sports they played, the desire to be healthier*, and *the desire to be competitive* (Fig. 4).

#### Avatar creations

All 59 boys had created a Current Avatar and a Preferred Avatar at the first data gathering time point. Two of the boys’ data from the avatar creations were removed. Their data could not be analyzed because the ATTAIN was mistakenly programmed to only include heights between 4’ 7” (55 in.) and 6′ 1″ (73 in.). These two boys entered a height of 4′ 4' and 4′ 6″, which exported zeros as measurements. The remaining 57 boys’ entries were included for analysis.

**Fig. 5:**
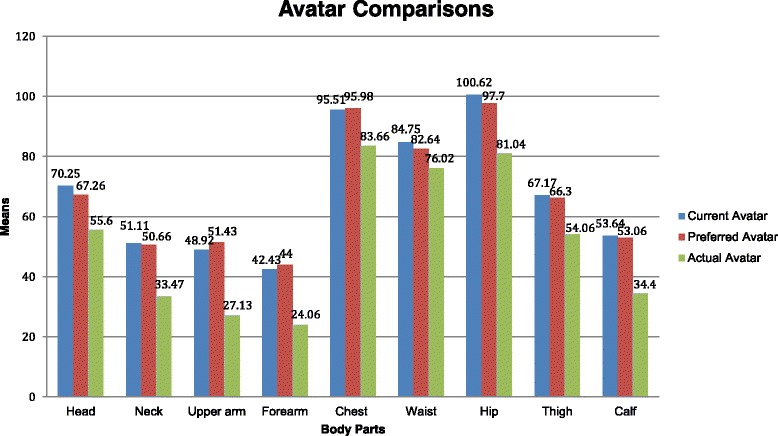
Means of body parts for each avatar

The means of each body part as a result of the boys creating their avatars and the measurements taken of the boys were computed (Fig. 5). When comparing the Current Avatar to the Preferred Avatar, the boys preferred to have bigger upper arms, forearms, and chests. On average their current and preferred perceptions of the other body parts were about the same. However a bigger difference occurred when comparing the Current Avatars and Preferred Avatars with the Actual Avatars. The boys indicated they thought they had bigger body parts and preferred to have bigger body parts when compared with their actual measurements. The widest difference existed when comparing the neck, upper arm, hips, and calves.

#### Internal consistency

Internal consistency was calculated using Cronbach’s alpha for the attitude items. The intentions/action items were not measured as continuous variables, therefore, were not included in the analysis. Whenever a participant responded to a certain item with *I do not want to answer,* his data were excluded. Of the 55 boys, 46 data were included for analysis. The attitude construct had a Cronbach’s alpha of 0.62, which was slightly below recommended levels [33].

#### Test-retest reliability

Two weeks following the first administration, 55 boys completed the ATTAIN. The boys were asked to answer the same 20 attitude survey items, 3 intention/action items, and recreate their Current Avatar and Preferred Avatar at this second time point. Intraclass correlation coefficients (ICC) were configured on the attitude items to assess for test-retest reliability (Table 3). If a participant responded with *I do not want to answer,* his data were excluded for that particular item. Of the 55 boys, 41 data were included for analysis. Test-retest reliability ICC (A,k) ranged from a low of 0.33 to a high of 1.00 for the attitude items and 0.70 for the overall attitude survey.

The boys were asked to recreate their Current Avatar and Preferred Avatar at the second time point. The ICC for test-retest reliability was calculated for each body part of the avatars (Table 3). The ICC (A,k) ranged from a low of 0.56 to a high of 0.76 for the Current Avatar computer-generated body part measurements from the first to second time points. For the Preferred Avatar the ICC (A,k) ranged from a low of 0.47 to a high of 0.83.

**Table 3:** Test re-test reliability of survey items and avatars

Item	Test Re-Test Reliability Intraclass Correlation	Response choices
*Attitude Overall Scale*	0.70	
*My…*		Too small, just right, or too big
Head/neck is	1.00	
Chest is	0.69	
Stomach is	0.74	
Arms are	0.63	
Butt is	0.80	
Legs/thigh are	0.33	
*I like my…*		Strongly disagree, disagree, agree or strongly agree
Head/neck	0.47	
Chest	0.77	
Stomach	0.84	
Arms	0.81	
Butt	0.80	
Legs/thighs	0.72	
*It is important for me to change my…*		
Head/neck	0.47	
Chest	0.64	
Stomach	0.82	
Arms	0.77	
Butt	0.41	
Legs/thighs	0.76	
*I am a good weight for my height.*	0.76	
*When you look at yourself, how do you describe your weight?*	0.97	Underweight, about right weight, a little overweight, overweight, or very overweight
*Current Avatar body parts*		Computer-generated by the boys
Head	0.56	
Neck	0.56	
Upper arm	0.73	
Forearm	0.69	
Chest	0.60	
Waist	0.74	
Hip	0.58	
Mid-thigh	0.76	
Calf	0.62	
*Preferred Avatar body parts*		Computer-generated by the boys
Head	0.52	
Neck	0.59	
Upper arm	0.83	
Forearm	0.58	
Chest	0.80	
Waist	0.51	
Hip	0.66	
Mid-thigh	0.47	
Calf	0.49	

The boys were asked to recreate their Current Avatar and Preferred Avatar at the second time point. The ICC for test-retest reliability was calculated for each body part of the avatars (Table 3). The ICC (A,k) ranged from a low of 0.56 to a high of 0.76 for the Current Avatar computer-generated body part measurements from the first to second time points. For the Preferred Avatar the ICC (A,k) ranged from a low of 0.47 to a high of 0.83.

## Discussion

The purpose of this paper was to describe the development and initial testing of the ATTAIN, an instrument to be used as a screening measure to capture boys’ attitudes toward their bodies, body parts, and weight. The instrument was based on the concepts of the IBM and included 23 survey items and avatar creation. Following the steps of instrument development, the ATTAIN was found to have high content validity, slightly below recommended levels for internal consistency, and varied test-retest reliability. The boys’ ideas about attitudes revealed interesting findings that we think are among the first to be reported.

Boys’ attitudes about their bodies varied. There were boys who did not like certain body parts; thought it important to change their body parts; and indicated, using their Preferred Avatar, that they wanted smaller or bigger body parts. More than half the boys wanted to change their bodies as indicated by the responses they gave for the attitude items. Yet almost three-quarters indicated they were a good weight for their height. For most of these boys, they were not trying to change their weight but instead gain muscle, because they were participating in sports or had inner feelings of competitiveness as opposed to being dissatisfied with their outward appearance or how others perceived them. This finding is similar to Bearman and colleagues, [20] who suggested that one’s perception of gaining or losing weight may be of greater psychological relevance than one’s physical dimensions. The boys gave responses to the attitude items about what they did and didn’t like and whether it was important to change or not change certain body parts; however, when creating the Current and Preferred Avatars the boys did not indicate a major change between the two avatars. They believed they wanted to change their body parts but did not demonstrate it using the physical representation of the avatars.

The ATTAIN items focused on how boys felt about their bodies and body parts. The avatars were used in conjunction with the items to further document the boys’ likes/dislikes and importance of changing certain body parts. Consistent with the recommendation made by Brener et al. [21] to provide a standard, the ATTAIN provided the boys with a personal standard of how they perceived their current bodies and how they preferred their bodies to be. The survey items and avatars afforded a detailed and comprehensive report of the boy’s perceptions, allowing for future discussions with parents, researchers, and health professionals such as primary care providers and school nurses on changing his body and body parts.

Our findings extend those of Yan and colleagues [34] who reported that adolescent boys misperceived their weight status by misclassifying their weight status (i.e. underweight when healthy weight, or healthy weight when overweight). The boys tended to overestimate their body parts when their actual measurements were compared to their Current Avatar measurements. When comparing their BMI-for-age percentile weight category to how they described their weight there were boys who overestimated and underestimated their weight category.

There were varied perceptions as to how they preferred their body parts to be. They preferred to have bigger body parts as indicated by the difference between the Actual Avatar and the Preferred Avatar. As Bearman and colleagues [20] reported, adolescent boys associate body satisfaction with gaining muscle. These boys may have wanted more muscular body parts by designating a preference for bigger body parts.

The attitude items had an internal consistency slightly below recommended levels. The responses from the survey items and avatar creations used in the ATTAIN from baseline and 2 weeks later generated varied stability. These findings may be the result of the topics of weight and body image as something that is rarely discussed by boys. This may have been the first time this sample of boys had expressed their thoughts about themselves using the survey items and avatars. The boys may have reflected upon their answers and when they responded to the same items 2 weeks after the first, they may have seen their bodies, body parts, and weight in a different way and indicated such with different responses.

### Limitations

While this study is original and innovative, it is preliminary, and the findings must be interpreted within its limitations. The sample for this study was small, limiting the statistical tests we could use, and comprised predominantly of Caucasian/White rural boys. Partnering with schools, boys’ clubs, primary care institutions, and other agencies for long-term strategies to reduce overweight and obesity would improve racial/ethnic representation and sample size. Eliciting from the boys what they were thinking as they responded to the items and created the avatars would provide more depth to our understanding of their responses. The age range was also a limitation, as only young adolescent boys were targeted. The researcher would need to conduct further testing with older adolescents to determine the differences in their attitudes toward their bodies, body parts, and weight.

### Future research

It would be beneficial to conduct more focus groups with boys, school nurses, clinic providers, public health nurses, parents, and informational technologists to provide a better awareness of how to further enhance the ATTAIN. The ATTAIN will need to be tested further with a large sample of boys this age and other ages as well as of boys of diverse races and ethnicities. There is a need for further research to adapt the ATTAIN to accommodate its findings and to establish discriminant and convergent construct validity [35].

### Use as a screening instrument

The ATTAIN has potential to be used a screening instrument because it does not require a prescription from a health care provider. It also can be administered by trained professionals in a short period of time to assess adolescent boys’ perceptions of themselves including their bodies, body parts, and weight and appropriately counsel and refer them. Nurses can also collaborate with parents, registered dieticians, exercise therapists, and school administrators to eventually screen all adolescents. As the ATTAIN is further improved, it may help screen young adolescent boys by providing information as to what changes they want to make to their bodies.

This is one of the first studies to begin looking at the difference in perceptions using individual body parts in addition to weight. Using the survey items and avatars from the ATTAIN can be used to screen adolescents and provide an assessment of accurate perceptions and preferences the boys have about certain body parts. Primary care remains underutilized for providing preventive services [36] and offers a potentially instrumental setting to assess obesity risk for all children and adolescents while providing guidance on health behaviors to minimize the risk [13] and referring to appropriate interventions.

## Conclusion

The result of this study is a theoretically derived instrument with appropriate content for young adolescent boys and variable reliability. The attitude construct, as measured by the ATTAIN, was meaningful to the boys. The ATTAIN was found to have high content validity, slightly below recommended levels for internal consistency and varied stability. The long-term goal of the ATTAIN is to make it available to researchers and professionals to screen and focus on young adolescent boys’ understanding of their attitudes toward their bodies to implement appropriate targeted interventions that help them attain and maintain healthy bodies. The ATTAIN will require continued development and testing to establish construct and discriminant validity.
